# Should smoke exposure be considered an etiologic factor for cardiovascular disease?

**DOI:** 10.4155/fsoa.2015.0018

**Published:** 2016-01-28

**Authors:** Aurelio Leone

**Affiliations:** 1 Fellow of the Royal Society for Promotion of Health (FRSPH), UK; 2 Fellow of the American Society of Hypertension, USA; 3 Via Provinciale 27, 19030 Castelnuovo Magra, Italy

**Keywords:** blood vessels, etiologic factor, heart, smoking

It is commonly believed and nearly completely accepted that cigarette smoking is a major cardiovascular risk factor for the heart and blood vessels because of the effects of some compounds, primarily nicotine and carbon monoxide, which are able to harm the cardiovascular system [1–5]. As a result of this, even passive smoking was included by the American Heart Association in their group of cardiovascular risk factors for both adolescents and adults [2].

Ischemic heart disease and endothelial dysfunction are the main effects observed as a result of both types of smoking – active and passive smoke exposure [6–9]. These changes have been clearly documented by clinical, pathological and experimental findings and, although they are of a different degree and extent in various reports, are reproducible in the same standardized procedures [10–12]. Functional, but initially reversible, and structural alterations [11,12], which become over the years firm lesions [13,14], may often be seen as a consequence of nicotine and carbon monoxide activity.

Briefly, impaired endothelium-dependent vasodilation due to a decrease in nitric oxide availability; reduced tolerance to exercise in both healthy individuals and individuals suffering from ischemic heart disease; as well as enhanced heart rate and systolic blood pressure due to sympathetic nervous system stimulation, carboxyhemoglobin and increased catecholamine release, are the transient and functional responses observed during acute exposure to smoking. On the other hand, chronic exposure to tobacco smoke causes well-established pathological disorders, typically due to carbon monoxide, consisting of myocardial lesions related to ischemic heart disease, and smoke cardiomyopathy with intracellular and ultrastructural alterations, primarily identified in experimental animals [14]. These lead to heart failure of various degrees, which mainly depends on the duration of the exposure. Moreover, vascular alterations of a degenerative type, which develop into atherosclerosis and its complications, affect primarily coronary, carotid and cerebral arteries. In addition, it is worth noting that microcirculation, because of the structure of resistance arteries, shows arteriosclerosis with enhanced vascular tone, which is the result of the anatomical lesions in the artery wall characterized by elastic fiber fragmentation and collagen deposits [15]. Increased arterial stiffness has also been documented in heavy smokers [16].

Therefore, it is worth noting that six markers often may change as a consequence of smoking exposure: endothelium with endothelial dysfunction; artery wall with lesions of different degrees including up to atherosclerotic plaque formation; blood pressure, which increases systolic values; heart rate, which closely follows blood pressure characteristics; myocardial function impairment and structural alterations of myocardial fibers leading up to necrosis.

Smoking compounds exert cardiovascular damage by direct and mediated mechanisms involving sympathetic and adrenergic stimulation, increased blood carboxyhemoglobin concentrations and cellular hypoxia [17].

By carefully analyzing these data, a fundamental concept emerges: cigarette smoking always causes the same damage to the heart and blood vessels in all subjects exposed, although the characteristics and type depend on various factors, primarily duration of the exposure to smoking toxics. In addition, there is evidence that a strongly adverse relationship, which is undoubtedly above a mere risk, exists between smoking and the cardiovascular system.

A risk factor [18] is associated with the underlying disease with a statistical, but probabilistic relation. The risk is expressed through a statistical estimate that, as it becomes closer to 100% – which, however, never can be reached – as a higher occurrence of the event related is demonstrated. Removing the risk factor can dramatically reduce, but not completely abolish the rate of the underlying disease since other, not always well-assessed conditions often contribute to its maintenance.

On the contrary, an etiologic factor [19] recognizes a link of cause–effect with the underlying disease, taking into account, however, that the occurrence of the disease is dependent on the power and potential charge of the pathogenic agent. When it reaches levels able to determine a disease, this event will always be seen with the same, even if of a different degree, type of alterations. So for example, when, in an infective disease with known etiology the causative micro-organism reaches a bacterial charge able to develop the disease, it always will occur.

It is worth noting, as aforementioned, that cigarette smoking should be considered an etiologic rather than a major cardiovascular risk factor. Thus smoking leads, in time, to the occurrence of the same cardiovascular disease, but with different degrees of changes. These are dependent on several factors such as number of cigarettes smoked, duration of exposure and health characteristics of the individual, among others. However, in pathological findings [19–21], when the experimental procedures are the same and well standardized, the cardiovascular damage from smoking is always reproducible in its features.

Finally, it is worth noting that cigarette smoking itself is able to cause a toxic disease whose etiologic factor should be considered the tobacco with its chemical compounds [22].

It is the author's personal opinion that a real link of cause–effect may certainly be identified between cigarette smoking and cardiovascular lesions, primarily ischemic heart disease and endothelial dysfunction, allowing tobacco smoke to be undoubtedly considered an etiologic factor of cardiovascular disease.
